# Continuous assessment and research on enhanced blood pressure control – CareBP study

**DOI:** 10.3389/fcvm.2026.1857454

**Published:** 2026-08-04

**Authors:** Sabina Schlottau, Stephanie Läer, Willi Cawello

**Affiliations:** Institute of Clinical Pharmacy and Pharmacotherapy, Heinrich Heine University Düsseldorf, Düsseldorf, Germany

**Keywords:** blood pressure profile, cuffless blood pressure monitoring, healthcare professionals, hypertension, model, pharmacists, prevention

## Abstract

**Introduction:**

Hypertension and its pre-stages are the leading cardiovascular risk factors worldwide, why awareness and prevention are highly relevant. Novel blood pressure devices can provide a broader picture at an earlier stage and enable an assessment of elevated values. The aim of this pilot study was to provide pharmacist-led support for the use, analysis and communication of a 14-day-cuffless blood pressure monitoring in potential hypertensive adults.

**Methods:**

For this pilot study ten adults were enrolled, who had elevated blood pressure values (>130 mmHg systolic and/ or >85 mmHg diastolic) in the past but no current cardiovascular diagnosis or medication. Participants were guided to use a cuffless blood pressure device (Aktiia/ Hilo bracelet) for 14 full days. The blood pressure profiles were analyzed with a previously developed blood pressure model and compared with profiles of normotensive adults.

**Results:**

The blood pressure profiles demonstrated individual blood pressure patterns in all participants. Six of ten participants were above the recommended 24-hour-threshold of 135/85 mmHg, one was at the threshold and three were under the threshold during the study period. Besides the blood pressure levels, there were differences regarding variability, night-dips and number of measurements. Compared to normotensives, base blood pressure was higher in potential hypertensives. Finally, the results were communicated to the participants for further discussion with other healthcare professionals.

**Discussion:**

The combination of a cuffless device, model-based analysis and pharmacist support represents a promising translational approach for individualized blood pressure monitoring. The results of the CareBP study show the extended value of 14 days of individual blood pressure monitoring and underline the relevance of this approach for cardiovascular disease prevention.

## 1 Introduction

Hypertension is often referred to as ’silent killer’ as it mostly progresses without symptoms, but it is the leading cardiovascular risk factor worldwide (1–3). The number of adults (aged 30–79years) worldwide living with hypertension is estimated to be more than one billion (1, 4). About 19% of all deaths in 2019 were related to hypertension (1, 5). Although there are established antihypertensive treatment options, challenges remain in hypertension management and diagnosis (3). Of those adults with hypertension, almost 50% are unaware of their condition, 42% are undergoing treatment and 21% have their condition under control (1, 2). Comprehensive blood pressure monitoring, assessment of individual blood pressure characteristics and hypertension prevention are necessary for cardiovascular risk reduction.

For this study, the term ‘potential hypertensives’ was introduced. ‘Potential hypertensives’ refers to individuals who had self-reported elevated blood pressure values in the past but had no hypertension diagnosis and were not receiving antihypertensive medication at the time of enrolment in the study. Elevated blood pressure was defined according to the 2023 guidelines as >130 mmHg systolic and/ or >85 mmHg diastolic (6), This group of participants represents individuals at increased cardiovascular risk, linked to the existing evidence that pre-stages of hypertension are associated with an increased risk of progression to hypertension and related complications (7, 8). The increased risk for cardiovascular disease and organ damage makes prevention and education highly relevant (9, 10). This is where pharmacies can contribute, as pharmacist care can improve blood pressure and adherence through counseling and patient education (11–13).

The 2023 ESH (European Society of Hypertension) and 2024 ESC (European Society of Cardiology) guidelines for the management of hypertension give recommendations for blood pressure screening and diagnosis (6, 14). The standard clinical method for blood pressure screening is office blood pressure measurement, where standardized measurements are conducted at the physician's office (6, 14). When elevated blood pressure values occur, it is recommended to confirm blood pressure with either home blood pressure monitoring (HBPM) or ambulatory blood pressure monitoring (ABPM) with an upper arm cuff (6, 14). With HBPM the patient monitors their own blood pressure at home with a validated device, whereas ABPM describes blood pressure measurements taken continuously (every 15–30 min) for 24 h (15). To evaluate the ABPM-measurements, mean blood pressure values (24 h, daytime and nighttime) are used in practice (6, 14). The two guidelines differ in their classifications of office blood pressure but align with the threshold of hypertension (≥140/ 90 mmHg) (6, 14). Below the threshold of hypertension, the 2023 ESH guidelines define the categories optimal (<120/ <80 mmHg), normal (120–129/ 80–84 mmHg) and high-normal (130–139/85–89 mmHg) (6), whereas the 2024 ESC guidelines have the classifications non-elevated blood pressure (<120/ 70 mmHg) and elevated blood pressure (120/ 70- <140/ 90 mmHg) (14).

Novel, cuffless blood pressure monitoring devices have been developed in the last years (16–20). Cuffless devices enable blood pressure measurements without influence on everyday situations or sleep due to the inflation of the cuff (16–18). Many new devices are based on photoplethysmography (PPG), which is an optical method (19, 20). The principle of PPG is that the skin of the wrist is illuminated with a light-emitting diode and depending on the vascular volume changes, light is reflected to a photodiode (16, 20). The cuffless blood pressure measurement device used in this study is the bracelet Hilo by Aktiia (Aktiia SA, Neuchatel, CH), which is a class IIa medical device (21). The bracelet collects the optical PPG-data automatically and intermittently at rest (21). The data are transferred via Bluetooth to the app on the smartphone and from there to the company's cloud (20). The Hilo app provides blood pressure values calculated by Aktiia's algorithm (20). Currently, calibration of the bracelet with an upper arm cuff (delivered with the bracelet) is necessary every month (20, 22). The evaluation of blood pressure in the Hilo app is based on the 2023 ESH guidelines (23). Nevertheless, cuffless blood pressure devices are not recommended in the guidelines for diagnosis or therapy assessment yet (6, 14, 15). However, cuffless blood pressure devices play an increasingly important role for customers and in research, as they have the potential to transform blood pressure monitoring and management through assessment of individual blood pressure patterns and variability (16, 17, 19, 24).

The first studies with the Aktiia/Hilo bracelet were validation studies, including testing with different body positions (21, 25), skin tones as well as hair follicle density (26), in comparison with ABPM (27) and during surgery (28). Further studies were conducted, including one to compare the Aktiia/ Hilo bracelet with a 24-hour-ambulatory monitor (ABPM) during a 12-week cardiac rehabilitation program (29). Almeida et al. have shown that, despite systematic differences in nocturnal blood pressure, the Aktiia/ Hilo bracelet provides blood pressure values that are comparable to the ABPM values (29). The COOL-BP (Continual Versus Occasional Blood Pressure) study was carried out in a remote hypertension program, where patients wore the Aktiia/ Hilo bracelet and measured blood pressure with a cuff (HBPM) (30). Unlu et al. investigated the correlation between the bracelet and the HBPM-device, which was moderate in non-simultaneous daytime measurements during the same periods. Most participants indicated that they prefer the bracelet because of usability, convenience and number of measurements (30). Aktiia itself performed a study to compare a large PPG dataset with simultaneous cuff-measurements (22). Calibration data of Aktiia users between 2021 and 2023 were analyzed with various machine learning models. Sola et al. concluded that PPG-based models provided the best correlation with the cuff and that blood pressure can be estimated with optical markers (22). Cuffless blood pressure devices have already been used in several projects, but there is consensus in the literature that standardization and validation protocols are necessary to ensure safety and quality of cuffless devices (15, 17, 19, 24, 31). Further challenges include the large amount of data provided by cuffless devices and patients using the devices for self-monitoring without consulting healthcare professionals (32). To take full advantage of all data, the complexity of physiological and pathophysiological information and to ensure a proper handling, analysis, interpretation as well as communication of the results, participants of this study received cuffless blood pressure monitoring in combination with pharmacist support and a model-based analysis.

This pilot study investigating potential hypertensive adults using a cuffless blood pressure device addresses a relevant research gap by generating blood pressure profiles in the possible transition phase to disease. This enables the identification of blood pressure patterns and can enhance prevention strategies. Furthermore, the differences between potential hypertensive and normotensive adults are examined.

## 2 Materials and methods

### 2.1 Study population

Before the start of this pilot study, ethical approval was obtained from the ethics committee of medical faculty of Heinrich Heine University Düsseldorf (Number: 2025-3247) and the study was registered in the German Clinical Trials Register (DRKS00037339). Participants were recruited using a flyer on the university campus, in a community pharmacy and through personal networks. All participants signed informed consent. Participants were included according to the following criteria (self-reported):

- Age 21–65 years
- Elevated blood pressure observed in the past (>130 mmHg systolic and/ or >85 mmHg diastolic)
- At the time of enrolment, no diagnosis of hypertension or other cardiac diseases
- At the time of enrolment, no antihypertensive medication
- Willingness to use smartphone and Hilo app.

### 2.2 Study design

**Figure 1:**
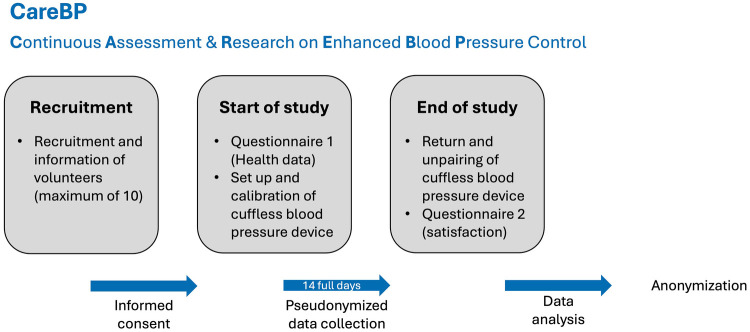
Overview of the CareBP study design.

At the beginning of the study (Figure 1) participants were asked to fill out a questionnaire about general and health-related information including lifestyle (Questionnaire 1, Supplementary Material). The questionnaire was developed considering questionnaires in the literature (33–35). This was followed up by a joint set up and calibration of the cuffless Aktiia/ Hilo blood pressure device and app with a pharmacist. From there, the bracelet was worn for full 14 days. During the wearing period, participants were asked to document sleeping times, activities and special events. At the end of the study, the bracelet was returned and unpaired. The collected data was analyzed and assessed by a pharmacist. Afterwards, the pharmacist communicated the individual blood pressure values and profiles with the participants and answered their questions. Both the data from the questionnaires and the blood pressure values were initially collected pseudonymized. Therefore, study IDs were assigned and only known to the study lead. This was necessary to contact participants in case of any concerns. After data analysis, the study ID list was deleted to anonymize the data.

### 2.3 Data analysis and profile modeling

Defined study endpoints were the blood pressure values over a period of two weeks (systolic and diastolic) as well as the participant satisfaction. For analyzing the blood pressure profiles, a previously developed blood pressure model was used. The blood pressure model is a non-linear regression model applied in the programming language R (36). This model was developed based on cuffless blood pressure measurements in normotensive adults over a period of 14 days (37). In the analysis of this study, the basic model was applied. Detailed information about the model development can be found in the corresponding publication (37). The blood pressure model uses exponential formula to describe the physiological circadian rhythm in blood pressure profiles. Starting from a basis blood pressure (base), the parameter incr describes the extent to which the blood pressure changes. The blood pressure change as a rate is described by the parameter k. In addition, the model includes the times t1 and t2, where t1 is defined as starting point for wake time and t2 as bedtime.

Depending on the phase of day, blood pressure is described by the following formula [t = time (h)]:

**Equation 1**. Wake phase, day (t1 < t < t2)

$$bp[i]=base+incr*(1-e^{-k*(t-t1)})\;$$

**Equation 2**. Bedtime, until midnight (t > t2)

$$bp[i]=base+incr*(1-e^{-k*(t2-t1)})*e^{-k*(t-t2)}\;$$

**Equation 3**. Bedtime, time between midnight and wake phase (t < t1)

$$bp[i]=base+incr*(1-e^{-k*(t2-t1)})*e^{-k*(t+24-t2)}$$

The respective model parameters were analyzed using descriptive statistics. Normal distribution of blood pressure values and model parameters was determined with Shapiro–Wilk test. To compare this study of potential hypertensives with normotensive adults, data of a previous study were taken and examined (38). As a statistic test Mann–Whitney-U-Test was used as the model parameters did not follow a normal distribution. The variability of blood pressure and its parameters was also considered, where intra-individual variability indicates the differences within a participant over time and inter-individual variability indicates the differences between the participants. For the assessment of blood pressure, the daytime ABPM threshold (135/ 85 mmHg) from the guidelines (6, 14) was used as a reference for contextualization. The decisive factor was the level of the blood pressure profile (the predicted values) and not single observed values. The evaluation of model performance was based on goodness-of-fit plots. Across all participants, predicted vs. observed values were plotted and time-dependence of weighted residuals was analyzed individually for each participant. All analyses were performed using R (version 4.5.1).

### 2.4 Satisfaction questionnaire

Finally, the satisfaction and perception questionnaire was filled out, where statements had to be rated on a 5-point-likert-scale with 1 indicating “strongly disagree” and 5 indicating “strongly agree” (Questionnaire 2, Supplementary Material). The questionnaire included statements on usability, comfort, application in healthcare and overall satisfaction.

## 3 Results

### 3.1 Study population

A total of ten participants with elevated blood pressure values in the past were enrolled for this study. All participants wore the cuffless Aktiia/ Hilo blood pressure device over a period of 14 full days. Most participants documented their sleeping times as well as special events. The health questionnaire (Questionnaire 1 at the beginning of the study) revealed that the lifestyles of the participants were varying with free time activities reaching from <75 to ≥150 min per week. The activity during work extended from only sitting to predominantly walking and standing. Most participants stated that they eat balanced with plant- and animal-based food, two participants eat predominantly animal-based food. More detailed information about the participants’ diet can be found in Supplementary Table 1. 50% of participants stated that they have family members (grandparents/ parents/ siblings) with diagnosed hypertension. 30% of participants reported that family members had suffered from cardiovascular events such as heart attacks or strokes. The participant characteristics are shown in Table 1.

**Table 1:** Participant characteristics.

Characteristics	Mean (± SD), range
Age (years)	42 (± 10), 28–59
Body-Mass-Index (BMI, kg/m^2^)	28.7 (± 3.6), 24.2–34.3
	Percentage (%)
Sex, percentage of men	70%
Smokers	10% currently, 50% in the past
Vegetarians	0%
Family members with hypertension (grandparents/ parents/ siblings)	50%
Family members with cardiovascular events (grandparents/ parents/ siblings)	30%
Diabetics	10%
Previous medical 24-hour measurement (ABPM)	50%

### 3.2 Participant history

Each participant reported individual motivations for joining a cuffless blood pressure monitoring, when they were informed about the study. Table 2 summarizes the participant histories and shows the context in which elevated blood pressure was observed in the past. The first (male, 28 years) and the second (male, 31 years) participant had both single elevated self-measured blood pressure values. Both participants visited their physician, but ABPM did not lead to a hypertension diagnosis, respectively. The first participant additionally described that he had increased nervousness and headaches in the months before the study. In the third participant (female, 59 years), single self-measured as well as office blood pressure values were elevated, but the ABPM did not confirm hypertension. In the fourth participant (male, 37 years), office and ambulatory blood pressure values were elevated years before the study. In response the patient changed his lifestyle, lost weight and exercised more. He managed to reduce his blood pressure so that ABPM was classified as normal, but uncertainties about the further development remained. The fifth participant (male, 49 years) had elevated office blood pressure approximately nine months before his study participation. The physician recommended daily measurements with a cuff (HBPM), an ABPM was not conducted. During HBPM, elevated blood pressure values were observed, but the participant wanted to wait and monitor further before taking medication. In the sixth (male, 53 years) and seventh participant (female, 46 years) single elevated office blood pressure values were measured but not followed up, respectively. No ABPM was carried out. The eighth participant (female, 34 years) had elevated blood pressure after pregnancy and birth (one year before the study) but returned to normal. As single office blood pressure values were elevated again, she was concerned that she might have hypertension. The ninth participant (male, 45 years), self-measured elevated blood pressure at home with the family. Medical check-ups had not been carried out for months. With the cuffless blood pressure measurements he wanted to gain clarity about his blood pressure. The tenth participant (male, 34 years, diabetic) had self-measured elevated blood pressure, the following ABPM did not lead to a hypertension diagnosis. He expressed major concerns about his blood pressure.

**Table 2:** Participant history and motivation for joining a cuffless blood pressure monitoring.

Participant	Sex	Age [Years]	ABPM	Participant history and motivation for joining a cuffless blood pressure monitoring
1	Male	28	Conducted, without diagnosis	- Single self-measured blood pressure values: elevated
				- Increased nervousness and headaches in the months before the study
2	Male	31	Conducted, without diagnosis	- Single self-measured blood pressure values: elevated
3	Female	59	Conducted, without diagnosis	- Single self-measured and office blood pressure values: elevated
4	Male	37	Conducted, follow-up classified as normal	- Office and ambulatory blood pressure values years before the study: elevated
				- In response: lifestyle changes, weight loss, exercise → blood pressure reduction
5	Male	49	No	- Office blood pressure values approx. nine months before the study: elevated
				- Recommendation of physician: HBPM
				- HBPM: elevated values
				- No follow-up; patient wanted to wait before taking medication
6	Male	53	No	- Office blood pressure values: elevated
				- Not followed up
7	Female	46	No	- Office blood pressure values: elevated
				- Not followed up
8	Female	34	No	- One year before the study: elevated blood pressure after pregnancy and birth
				- Blood pressure returned to normal
				- Office blood pressure: elevated
9	Male	45	No	- Single self-measured blood pressure values: elevated
				- No medical check-ups for months
10	Male	34	Conducted, without diagnosis	- Single self-measured blood pressure values: elevated

Office blood pressure describes blood pressure measurements in the physician's office. ABPM, Ambulatory blood pressure monitoring (24 h); HBPM, Home blood pressure monitoring

### 3.3 Blood pressure

During the CareBP study 5516 blood pressure values were recorded with the Aktiia/ Hilo bracelet. On average, these were 39 values per day per person. The number of measurements per person ranged from 438 to 733 in 14 days, depending for example on activities.

**Figure 2:**
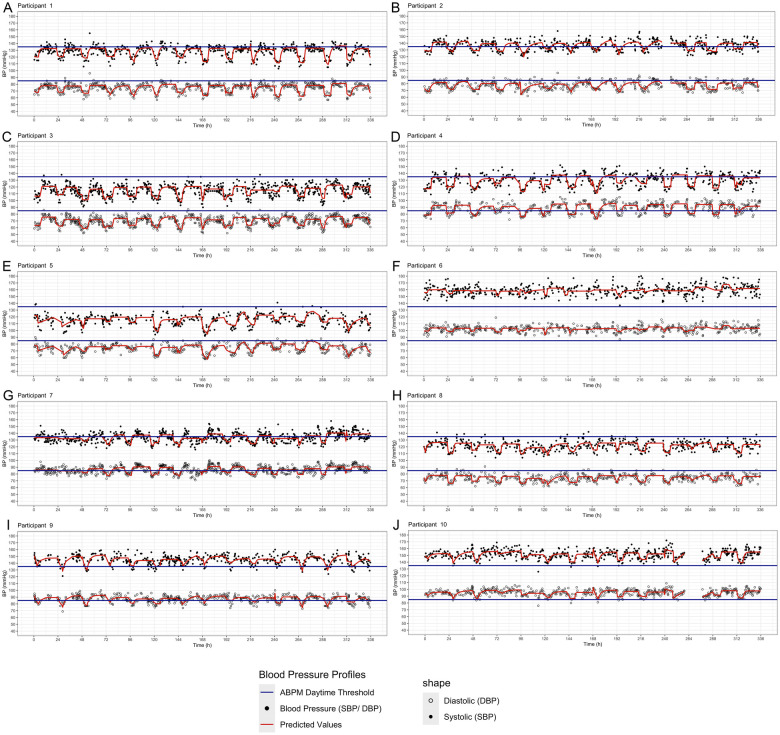
Blood pressure profiles of the ten participants **(A–J)** over a period of 14 days. The dots represent the observed blood pressure values, the red lines represent the predicted blood pressure profiles, and the blue horizontal lines are the ABPM daytime thresholds for hypertension (135/ 85 mmHg) according to the hypertension guidelines (6, 14).

The predicted blood pressure profiles and the observed values showed individual blood pressure patterns and ranges. Multiple participants were identified with elevated blood pressure that had not been detected in previous standard of care. Table 3 provides an overview of the observed blood pressure ranges as well as the predicted median blood pressure values with the corresponding assessment. Figure 2 visualizes that the blood pressure profiles for six of the ten participants were exceeding the ABPM reference daytime threshold of 135/85 mmHg (participants 2, 4, 6, 7, 9, 10). The blood pressure profile of participant 1 was at the threshold for the entire study period. In participant 4, especially the diastolic blood pressure values were exceeding the reference threshold. For participants 3, 5 and 8, the blood pressure profiles were below the reference threshold.

**Table 3:** Assessment of observed and predicted blood pressure values for all ten participants.

Participant	Blood pressure values: systolic/ diastolic [mmHg]			Assessment
	Observed (range, min-max)	Predicted (daytime median)	Predicted (nighttime median)	
1	103–155/56–96	134/78	115/64	Systolic at threshold
2	118–158/62–96	141/84	128/71	Systolic above threshold
3	90–138/52–87	120/74	106/63	Below threshold
4	109–152/72–105	136/93	120/80	Above threshold, especially diastolic
5	92–141/58–90	123/81	108/68	Below threshold, high variability
6	137–192/87–119	162/104	157/99	Above threshold
7	116–158/71–100	137/88	127/80	Above threshold
8	104–142/62–91	123/77	111/67	Below threshold
9	121–160/69–100	150/90	135/80	Above threshold
10	126–172/76–109	153/97	140/87	Above threshold

For assessment, the recommended daytime threshold of ambulatory blood pressure monitoring (ABPM) of 135/ 85 mmHg (6, 14) was applied as a reference on the predicted blood pressure values for contextualization of the blood pressure profiles.

### 3.4 Model parameters

**Figure 3:**
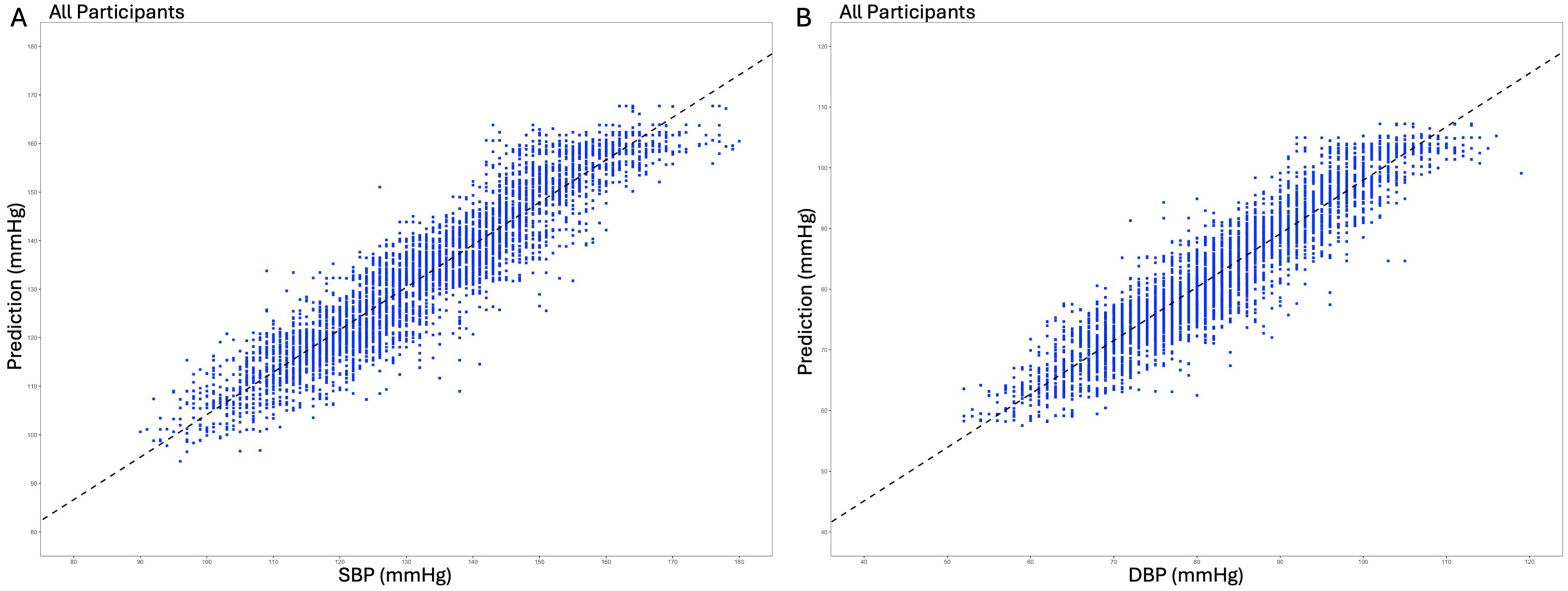
Representation of model performance in potential hypertensives based on the predicted and observed values for systolic blood pressure **(A)** and diastolic blood pressure **(B).**

For participant 1 the blood pressure model predicted base-ranges of 102–121/ 53–68 mmHg and incr-ranges of 11–30/ 9–24 mmHg (Tables 4, 5). The median parameters of 115/ 64 (base) and 18/ 15 mmHg (incr) indicated that the average blood pressure of participant 1 was approximately 133/ 79 mmHg (base+incr) during the day and approximately 115/ 64 mmHg (base) during the night. Although the values for participant 5 were predominantly below the reference threshold, the blood pressure profile showed a high variability. The blood pressure fluctuated more in participant 5 than in 3 and 8, which is underlined by the high range and high interquartile range of the parameter incr (Tables 4, 5). Participant 6 had the highest blood pressure values and the least pronounced night-dip (underlined with smallest incr-median in systolic and diastolic blood pressure while having the highest blood pressure base). A small interquartile range for parameter base in each participant (intra-individual variability) shows that the middle 50% of values were closely clustered around the median, whereas the interquartile range overall (inter-individual variability) was higher. These differences in intra- and inter-individual variability did not occur for parameters incr and k. The visualization of predicted vs. observed blood pressure values (Figure 3) indicated an overall consistency without systematic deviations for systolic and diastolic blood pressure. The distribution of weighted residuals over time without systematic patterns (Supplementary Figures 2, 3) represented that there was no time dependence of weighted residuals.

**Table 4:** Descriptive statistics of model parameters for systolic blood pressure (base = basic blood pressure, incr = extent of blood pressure changes, k = change rate, t1 = wake time, t2 = bedtime).

Participant	Median with range (minimum - maximum)					Interquartile range		
	base [mmHg]	incr [mmHg]	k	t1	t2	base	incr	k
1	115.3 (102.1–121.0)	18.4 (10.5–30.1)	0.93 (0.42–2.00)	5.24 (2.52–8.08)	22.89 (21.34–24.6)	6.3	7.0	1.4
2	127.7 (116.2–132.2)	12.8 (8.7–24.8)	0.53 (0.11–2.00)	5.16 (1.00–7.92)	22.57 (19.33–23.87)	3.6	9.7	1.2
3	106.3 (98.0–115.4)	13.5 (0.0–22.8)	1.86 (0.2–2.00)	5.22 (1.12–6.27)	20.66 (18.29–23.12)	6.7	5.0	0.9
4	120.3 (106.5–125.7)	15.9 (8.0–30.8)	1.51 (0.36–2.00)	6.02 (4.43–7.12)	21.94 (19.07–23.69)	5.8	6.1	1.2
5	107.7 (93.2–114.6)	15.7 (5.5–59.8)	0.71 (0.01–2.00)	4.43 (1.11–9.23)	21.9 (18.00–25.00)	8.4	13.3	1.7
6	156.6 (149.1–161.7)	5.1 (0.0–22.7)	0.4 (0.01–2.00)	3.64 (1.00–9.73)	22.11 (18.00–25.00)	4.1	8.0	1.7
7	126.8 (119.7–135.1)	10.5 (0.0–16.5)	1.15 (0.01–2.00)	3.61 (1.00–5.47)	20.32 (18.00–24.37)	8.2	9.5	1.6
8	111.3 (103.9–115.8)	11.9 (7.7–20.8)	2.00 (0.21–2.00)	4.89 (1.00–6.02)	23.10 (21.13–23.58)	4.2	3.8	1.5
9	135.0 (125.7–140.6)	14.7 (6.5–26.8)	0.37 (0.06–2.00)	4.46 (1.93–5.83)	23.00 (18.00–25.00)	7.5	12.8	0.3
10	140.1 (136.4–151.9)	12.9 (3.6–60.0)	0.45 (0.02–2.00)	5.64 (1.12–11.00)	23.71 (21.90–25.00)	5.9	9.7	1.3
Overall	122.6 (93.2–161.7)	13.2 (0.0–60.0)	0.83 (0.01–2.00)	4.65 (1–11.00)	22.36 (18.00–25.00)	23.2	8.9	1.6

**Table 5:** Descriptive statistics of model parameters for diastolic blood pressure (base = basic blood pressure, incr = extent of blood pressure changes, k = change rate, t1 = wake time, t2 = bedtime).

Participant	Median with range (minimum - maximum)					Interquartile range		
	base [mmHg]	incr [mmHg]	k	t1	t2	base	incr	k
1	63.6 (52.8–68.1)	14.8 (9.3–24.4)	0.85 (0.26–2.00)	4.87 (2.53–8.14)	22.46 (21.36–24.78)	4.2	5.3	1.4
2	70.8 (63.7–73.9)	13.3 (6.3–19.5)	0.52 (0.15–2.00)	5.64 (1.00–7.94)	22.47 (19.33–25.00)	4.2	6.4	1.5
3	62.8 (57.9–66.8)	11.5 (8.8–18.1)	1.17 (0.4–2.00)	5.14 (1.00–6.28)	20.56 (18.00–22.51)	5.0	3.7	0.9
4	80.3 (67.6–85.3)	13.0 (6.0–28.2)	2.00 (0.34–2.00)	6.18 (2.38–7.54)	22.17 (18.73–23.67)	4.5	3.7	1.0
5	67.5 (55.8–73.0)	13.0 (4.6–22.5)	0.66 (0.05–2.00)	4.59 (1.00–8.14)	21.34 (18.00–25.00)	7.8	9.9	1.1
6	99.1 (89.5–103.2)	4.7 (0.0–14.5)	1.92 (0.14–2.00)	4.64 (1.00–9.62)	21.72 (18.00–25.00)	2.9	1.3	1.2
7	79.5 (76.7–87.1)	8.6 (0.0–12.6)	1.90 (0.09–2.00)	3.92 (1.45–5.52)	20.32 (18.00–22.32)	4.3	3.6	1.1
8	67.2 (62.6–70.3)	9.8 (6.3–15.2)	1.93 (0.11–2.00)	4.94 (1.43–8.68)	22.7 (18.82–23.93)	3.2	2.5	1.3
9	80.3 (72.6–85.1)	9.6 (4.3–18.7)	0.44 (0.11–2.00)	4.41 (1.93–7.25)	23.24 (18.00–24.97)	5.9	9.1	0.2
10	87.2 (82.9–93.9)	9.5 (2.3–60.0)	0.38 (0.01–2.00)	5.29 (2.72–11.00)	23.67 (21.58–25.00)	3.9	7.4	1.0
Overall	73.1 (52.8–103.2)	10.1 (0.0–60.0)	1.07 (0.01–2.00)	4.89 (1.00–11.00)	22.14 (18.00–25.00)	17.7	7.2	1.6

### 3.5 Comparison of potential hypertensives with previous data of normotensives

**Figure 4:**
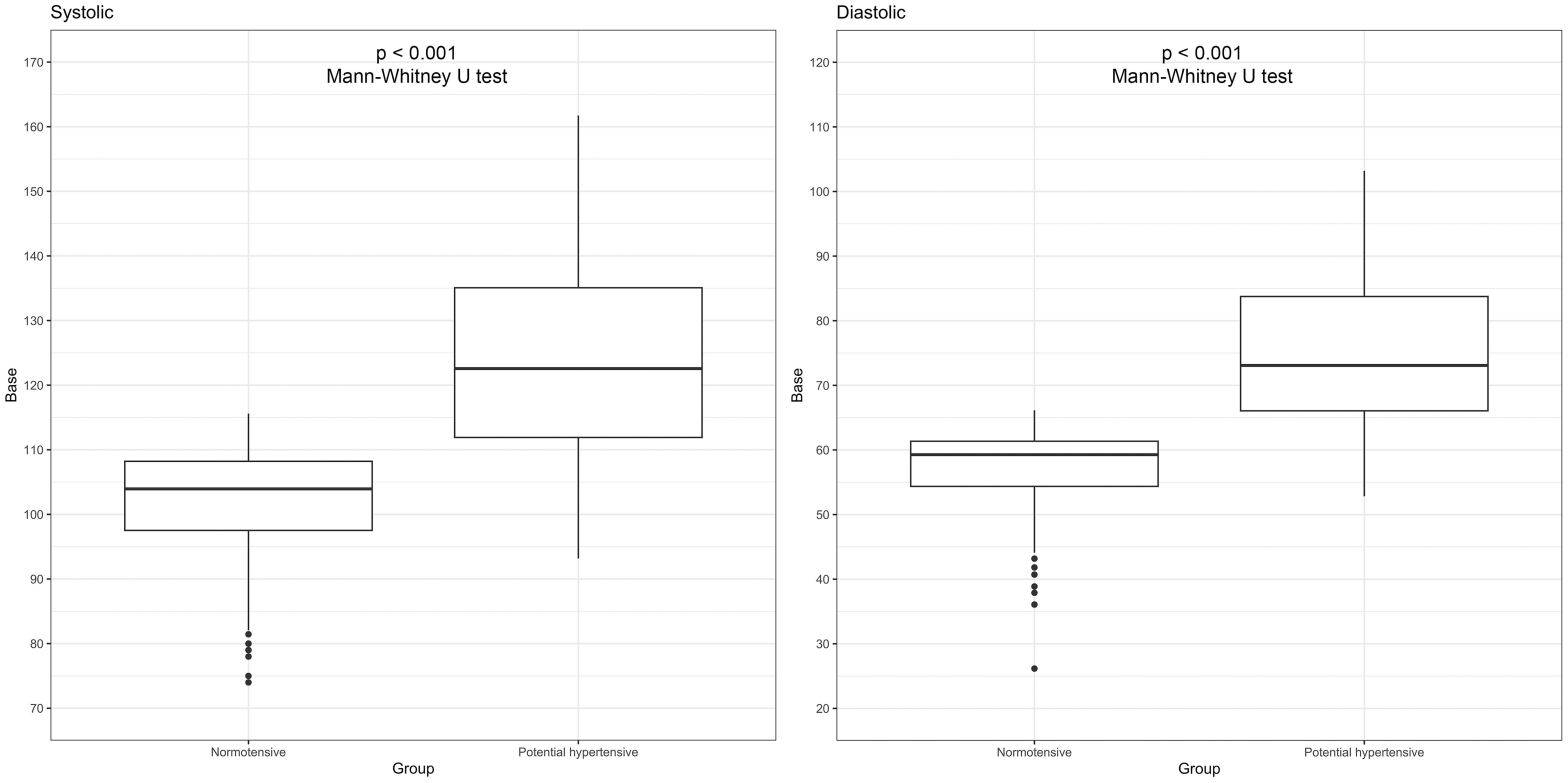
Comparison of model parameter base in potential hypertensives with already published data of normotensives (37, 38).

To observe whether there are differences between potential hypertensives and normotensives regarding blood pressure parameters, already published data were used (37, 38). In the already published pilot study (six normotensive adults) (38) as well as in the CareBP study (ten potential hypertensive adults) a cuffless blood pressure device (Aktiia/ Hilo bracelet) was worn for two weeks. Subsequently, a blood pressure model was developed (37) and applied to both study datasets. Figure 4 shows the comparison of the model parameter base between potential hypertensives and normotensives.

For the parameter base (Figure 4) a difference between the groups was observed (*p* < 0.001). For the parameters incr (*p* < 0.001 systolic and diastolic) and k (*p* < 0.001 systolic, *p* < 0.005 diastolic) there were also differences but less distinct. For wake time (t1) and bedtime (t2) there were no significant differences between the groups.

### 3.6 Satisfaction and perception

**Figure 5:**
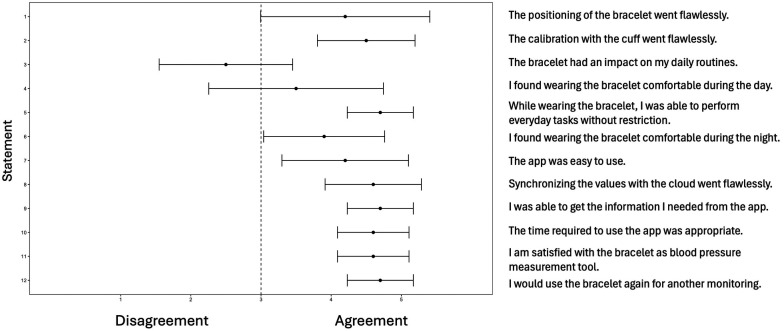
Forest-plot representing the participants’ satisfaction with the Aktiia/ Hilo bracelet. The dots represent the mean values and the horizontal bars the 95% confidence interval. The Likert scale (*x*-axis) was used from 1 = “strongly disagree” to 5 = “strongly agree”. The vertical dotted line corresponds to 3 as the neutral position.

Participant satisfaction with the Aktiia/ Hilo bracelet is presented in Figure 5, where all statements are listed alongside their respective results (Questionnaire 2 at the end of the study). The participants stated that the calibration went flawlessly, that they were able to perform everyday tasks without restriction, were satisfied with the app and would use the bracelet again for another monitoring. Some participants experienced the positioning, the impact on daily routines and the comfort during the day with limitations, resulting in no consensus in statements 1, 3 and 4.

Part of the questionnaire 2 was also the perception regarding blood pressure. The participants were asked whether they were able to develop an awareness for their blood pressure values, whether the study has been personally rewarding, whether the blood pressure monitoring helped to understand blood pressure changes better, if they have planned to continue blood pressure measurements (independently of the measurement method), if they would like reimbursement of continuous blood pressure monitoring by health insurance and how they evaluate the importance of pharmacist involvement in blood pressure monitoring. All six statements to perception of blood pressure were answered significantly with agreement (mean [95% CI]: 4.5 [3.98−5.02], 4.7 [4.23−5.17], 3.7 [3.04−4.36], 4.4 [3.71−5.09], 4.8 [4.39−5.21], 4.7 [4.23−5.17], respectively; Supplementary Table 2).

## 4 Discussion

The CareBP pilot study has provided blood pressure profiles based on cuffless blood pressure measurements for two weeks. The participants were potential hypertensives with elevated blood pressure values observed in the past but without cardiovascular diagnosis or medication at the time of study enrolment. The approach of this pilot study stands out by combining a cuffless device with a model-based analysis and professional guidance from a pharmacist. Furthermore, this study addresses a gap in research by collecting and analyzing data during the possible transition phase from normotension to hypertension, providing strong innovation and translational potential for prevention strategies. To the authors’ knowledge, this is the first time that this population of potential hypertensives has been examined in this way.

The main findings of the presented study are: (1) The generated blood pressure profiles show notable heterogeneity within the study population. (2) Both the occurrence of (highly) elevated and normotensive values in potential hypertensives underline the limited reliability of single blood pressure measurements. Half of the participants took an ABPM, but it is limited to 24 h and not seamlessly integrated in daily routines. A cuffless blood pressure device like the Aktiia/ Hilo bracelet provides a more comprehensive image of blood pressure. (3) The CareBP study confirms the findings from other studies that elevated blood pressure or hypertension can be masked and stay undiagnosed (2, 39). But this study also shows that just because elevated levels were detected at times, this does not necessarily mean that hypertension is present (participants 3, 5, 8).

For data analysis and visualization, a previously developed blood pressure model was used (37). Specifically, the basic model was applied, as more appropriate predictions were expected. While the extended model delivered good results in normotensives, it is more sensitive to unexpected patterns (e.g., missing night-dips). The basic model makes stable blood pressure predictions, is more compact in its structure and therefore more suitable for different datasets. The models were developed and initially tested with data from young and normotensive adults (age 22–29 years, BMI 19.8–30.8 kg/m^2^; Supplementary Figure 1) (38). As the participants of the CareBP study were individuals who had self-reported elevated blood pressure values in the past, either at home or at the physician's office, the predictions had to be suitable for heterogenous data. Based on the presentation of the predicted vs. observed values and the weighted residuals over time, it was determined that the basic model could adequately describe the blood pressure for potential hypertensives as well. Compared to normotensives, the base blood pressure is notably higher in potential hypertensives. The parameter base appears to be more individual than the parameters incr and k. In potential hypertensives inter-individual variability of parameter base was notably larger than intra-individual variability. Due to the variable nature of blood pressure, single elevated values were not considered for the evaluation of blood pressure levels. Instead, the recommended ABPM daytime threshold of 135/ 85 mmHg (6, 14) was applied to the predicted blood pressure profiles. It should be emphasized that in this study the ABPM threshold was not applied for diagnostic classification, but as a reference to support the contextual interpretation of the blood pressure profiles. In cases of elevated blood pressure values, the individuals were referred to their doctor. Some of the participants reported that they intended to consult their physician based on the observed blood pressure profiles and described their motivation for lifestyle-changes. After the end of the study, two participants also shared the information that they visited their physician, which resulted in antihypertensive therapy.

It is known that there are many factors influencing blood pressure - like the circadian rhythm (40, 41), the environment (42, 43), the socioeconomic status (44) and the lifestyle including diet (45, 46), activity (47, 48) and sleep (49, 50). Studies have also shown that the risk for hypertension and cardiovascular disease is increased with family history of hypertension or cardiovascular disease in first degree relatives as well as grandparents (51–54). Accordingly, information about lifestyle and medical history as well as the family history of cardiovascular diagnoses are required for risk assessment. For this reason, a health questionnaire (questionnaire 1) was completed at the beginning of the study. Diabetes mellitus can also be a risk factor for hypertension (10), but there are only few studies with cuffless blood pressure devices including diabetics (55–57). Until now, diabetics were excluded in studies using the Aktiia/ Hilo bracelet (25). In the described CareBP study a diabetic patient was included as only cardiovascular diagnoses were excluded. While the Aktiia/ Hilo set up, a calibration was conducted with an upper arm cuff like in all participants. During the study the diabetic participant became uncertain about his blood pressure values, as some of his self-measured values with a cuff deviated from the cuffless values. To address the concerns and clarify the situation, an additional blood pressure monitoring with a cuff in combination with the cuffless device was carried out at the study institute by a pharmacist. The reference measurements confirmed the data collected during the study. This additional data was not included in the study evaluation, so that the study results remain unaffected. As the participant's assumption about elevated blood pressure was supported by the study, the participant was referred to his physician.

This study has limitations regarding the measurement method and the size of the study population. For recording blood pressure, a cuffless device (Hilo by Aktiia) was used, which is a class IIa medical device (21), but not recommended in the ESH and ESC guidelines at this time (6, 14). A previous study suggests that night-dips are less pronounced in cuffless blood pressure devices than in ABPM (29). Accordingly, application of this monitoring in clinical practice is limited, yet. Since there are no established thresholds for cuffless devices yet, the recommended ABPM threshold was used as a reference to create a comparable framework for describing blood pressure profiles within the study population. This underlines the need for the harmonization of device-specific thresholds in the future. To encourage patients to monitor their blood pressure with guidance in the future as well, it is important that healthcare professionals are informed and integrated. The authors have shown that cuffless blood pressure monitoring can be performed under guidance of a pharmacist. As this is a pilot study with a small sample size, results cannot be generalized, but it provides fast, cost-effective and practical information about feasibility, data quality and initial indications of potential effects that require confirmation in larger studies. The results of this study indicate the potential of blood pressure profiles for screening and prevention.

In summary, the results of the study highlight the additional value that blood pressure profiles offer through cuffless devices. Single blood pressure values may not allow a complete and individualized assessment, so that under- or overdiagnoses of hypertension may occur. The multifactorial and dynamic nature of hypertension makes early and comprehensive screening a priority to enable targeted and individualized prevention and lifestyle modifications. Therefore, implementation of cuffless blood pressure monitoring with counselling in healthcare is required, with pharmacies offering expertise and a promising platform. The authors are convinced that this study holds practical significance for early hypertension intervention and provides a foundation for further research, screening and prevention projects.

## Data Availability

The raw data supporting the conclusions of this article will be made available by the authors, without undue reservation.

## References

[1] World Health Organization. Global Report on Hypertension: The Race Against a Silent Killer. Geneva (2023). Licence: CC BY-NC-SA 3.0 IGO.

[2] Kario K Okura A Hoshide S Mogi M. The WHO global report 2023 on hypertension warning the emerging hypertension burden in globe and its treatment strategy. Hypertens Res. (2024) 47(5):1099–102. 10.1038/s41440-024-01622-w 38443614

[3] Mills KT Stefanescu A He J. The global epidemiology of hypertension. Nature Reviews Nephrology. (2020) 16(4):223–37. 10.1038/s41581-019-0244-2 32024986 PMC7998524

[4] Zhou B Carrillo-Larco RM Danaei G Riley LM Paciorek CJ Stevens GA. Worldwide trends in hypertension prevalence and progress in treatment and control from 1990 to 2019: a pooled analysis of 1201 population-representative studies with 104 million participants. Lancet. (2021) 398(10304):957–80. 10.1016/S0140-6736(21)01330-1 34450083 PMC8446938

[5] Murray CJL Aravkin AY Zheng P Abbafati C Abbas KM Abbasi-Kangevari M. Global burden of 87 risk factors in 204 countries and territories, 1990–2019: a systematic analysis for the global burden of disease study 2019. Lancet. (2020) 396(10258):1223–49. 10.1016/S0140-6736(20)30752-2 33069327 PMC7566194

[6] Mancia G Kreutz R Brunström M Burnier M Grassi G Januszewicz A. 2023 ESH guidelines for the management of arterial hypertension the task force for the management of arterial hypertension of the European society of hypertension: endorsed by the international society of hypertension (ISH) and the European renal association (ERA). J Hypertens. (2023) 41(12):1874–2071. 10.1097/HJH.0000000000003480 37345492

[7] Khosravi A Gharipour M Nezafati P Khosravi Z Sadeghi M Khaledifar A. Pre-hypertension, pre-diabetes or both: which is best at predicting cardiovascular events in the long term? J Hum Hypertens. (2017) 31(6):382–7. 10.1038/jhh.2016.42 27334522

[8] Guo X Zhang X Guo L Li Z Zheng L Yu S. Association between pre-hypertension and cardiovascular outcomes: a systematic review and meta-analysis of prospective studies. Curr Hypertens Rep. (2013) 15(6):703–16. 10.1007/s11906-013-0403-y 24234576

[9] Kanegae H Oikawa T Kario K. Should Pre-hypertension be treated? Curr Hypertens Rep. (2017) 19(11):91. 10.1007/s11906-017-0789-z 29046988

[10] Jang I. Pre-Hypertension and its determinants in healthy young adults: analysis of data from the Korean national health and nutrition examination survey VII. Int J Environ Res Public Health. (2021) 18(17):9144. 10.3390/ijerph18179144 34501734 PMC8431073

[11] Gastens V Tancredi S Kiszio B Del Giovane C Tsuyuki RT Paradis G. Pharmacists delivering hypertension care services: a systematic review and meta-analysis of randomized controlled trials. Front Cardiovasc Med. (2025) 12:1–12. 10.3389/fcvm.2025.1477729 40161392 PMC11949927

[12] Morgado MP Morgado SR Mendes LC Pereira LJ Castelo-Branco M. Pharmacist interventions to enhance blood pressure control and adherence to antihypertensive therapy: review and meta-analysis. Am J Health Syst Pharm. (2011) 68(3):241–53. 10.2146/ajhp090656 21258029

[13] Reeves L Robinson K McClelland T Adedoyin CA Broeseker A Adunlin G. Pharmacist interventions in the management of blood pressure control and adherence to antihypertensive medications: a systematic review of randomized controlled trials. J Pharm Pract. (2021) 34(3):480–92. 10.1177/0897190020903573 32067555

[14] McEvoy JW McCarthy CP Bruno RM Brouwers S Canavan MD Ceconi C. 2024 ESC guidelines for the management of elevated blood pressure and hypertension: developed by the task force on the management of elevated blood pressure and hypertension of the European Society of Cardiology (ESC) and endorsed by the European Society of Endocrinology (ESE) and the European stroke organisation (ESO). Eur Heart J. (2024) 45(38):3912–4018. 10.1093/eurheartj/ehae178 39210715

[15] Jones DW Ferdinand KC Taler SJ Johnson HM Shimbo D Abdalla M. 2025 AHA/ACC/AANP/AAPA/ABC/ACCP/ACPM/AGS/AMA/ASPC/NMA/PCNA/SGIM guideline for the prevention, detection, evaluation and management of high blood pressure in adults: a report of the American College of Cardiology/American Heart Association joint committee on clinical practice guidelines. Hypertension. (2025) 82(10):e212–316. 10.1161/HYP.0000000000000257 40811516

[16] Berlot AA Berman J Borad A Mason TD Petriceks A Juraschek S. Remote blood pressure monitoring: a comprehensive review. Am J Hypertens. (2025)39:188–97. doi: 10.1093/ajh/hpaf097. 10.1093/ajh/hpaf097 PMC13271084 40452303

[17] Hu J-R Park DY Agarwal N Herzig M Ormseth G Kaushik M. The promise and illusion of continuous, cuffless blood pressure monitoring. Curr Cardiol Rep. (2023) 25(10):1139–49. 10.1007/s11886-023-01932-4 37688763 PMC10842120

[18] Li B Huang Y Mao W Liu J Ma Q. Applications and prospects of digital health technologies in cardiovascular nursing: smart devices, remote monitoring, and personalized care. J Multidiscip Healthc. (2025) 18:6275–86. 10.2147/JMDH.S548258 41059086 PMC12499597

[19] Kario K Asayama K Arima H Matsumoto C Nakagawa N Nomura A. Digital hypertension - what we need for the high-quality management of hypertension in the new era. Hypertens Res. (2025) 48:3130–46. 10.1038/s41440-025-02307-8 41028224

[20] Sola J Bertschi M Krauss J. Measuring pressure: introducing oBPM, the optical revolution for blood pressure monitoring. IEEE Pulse. (2018) 9(5):31–3. 10.1109/MPUL.2018.2856960 30273141

[21] Sola J Vybornova A Fallet S Polychronopoulou E Wurzner-Ghajarzadeh A Wuerzner G. Validation of the optical aktiia bracelet in different body positions for the persistent monitoring of blood pressure. Sci Rep. (2021) 11(1):20644. 10.1038/s41598-021-99294-w 34667230 PMC8526831

[22] Sola J Arderiu A Almeida TP Fallet S Yazdani S Haddad S. The quest for blood pressure markers in photoplethysmography and its applications in digital health. Front Digit Health. (2025) 7(1518322):1–11. 10.3389/fdgth.2025.1518322 40370706 PMC12075524

[23] How is Time in Target Range (TTR) calculated? Available online at: https://support.aktiia.com/en/articles/697294-how-is-time-in-target-range-ttr-calculated (Accessed 19 June 2026).

[24] Schutte AE Kollias A Stergiou GS. Blood pressure and its variability: classic and novel measurement techniques. Nat Rev Cardiol. (2022) 19(10):643–54. 10.1038/s41569-022-00690-0 35440738 PMC9017082

[25] Theiler K Sola J Damianaki A Pfister A Almeida TP Alexandre J. Performance of the aktiia optical blood pressure measurement device in the elderly: a comparison with double blinded auscultation in different body positions. Blood Press. (2023) 32(1):2281320. 10.1080/08037051.2023.2281320 37971487

[26] Vybornova A Polychronopoulou E Wurzner-Ghajarzadeh A Fallet S Sola J Wuerzner G. Blood pressure from the optical aktiia bracelet: a 1-month validation study using an extended ISO81060-2 protocol adapted for a cuffless wrist device. Blood Press Monit. (2021) 26(4):305–11. 10.1097/MBP.0000000000000531 33675592 PMC8248249

[27] Almeida TP Cortés M Perruchoud D Alexandre J Vermare P Sola J. Aktiia cuffless blood pressure monitor yields equivalent daytime blood pressure measurements compared to a 24-h ambulatory blood pressure monitor: preliminary results from a prospective single-center study. Hypertens Res. (2023) 46(6):1456–61. 10.1038/s41440-023-01258-2 37012424 PMC10239726

[28] Ghamri Y Axis A Braun F Pierrel N Solà J Schoettker P. Continuous optical blood pressure measurement in the perioperative setting (2018).

[29] Almeida TP Perruchoud D Alexandre J Vermare P Sola J Shah J. Evaluation of aktiia cuffless blood pressure monitor across 24-h, daytime, and night-time measurements versus ambulatory monitoring: a prospective, single-centre observational study. J Hypertens. (2025) 43(4):690–7. 10.1097/HJH.0000000000003960 39927495 PMC11872257

[30] Unlu O Cannon CP Lee S Gabovitch D Zelle D Chin N. Continual versus occasional blood pressure (COOL-BP) in remote hypertension management. Am J Hypertens. (2025) 38(5):295–302. 10.1093/ajh/hpaf003 39789184 PMC11997243

[31] Wehbe F Hiremath S. Cuffless blood pressure in 2025: from promise to practice: a narrative review. Curr Opin Nephrol Hypertens. (2026) 35(2):164–73. 10.1097/MNH.0000000000001150 41460057

[32] Cohen JB Byfield RL Hardy ST Juraschek SP Houston Miller N Mukkamala R. Cuffless devices for the measurement of blood pressure: a scientific statement from the American Heart Association. Hypertension. (2026) 83(3):e00254. 10.1161/HYP.0000000000000254 41376592 PMC13335599

[33] Craig CL Marshall AL Sjostrom M Bauman AE Booth ML Ainsworth BE. International physical activity questionnaire: 12-country reliability and validity. Med Sci Sports Exerc. (2003) 35(8):1381–95. 10.1249/01.MSS.0000078924.61453.FB 12900694

[34] Fuchs R Klaperski S Gerber M Seelig H. Messung der bewegungs- und sportaktivität mit dem BSA-fragebogen: eine methodische zwischenbilanz. Zeitschrift für Gesundheitspsychologie. (2015) 23(2):60–76. 10.1026/0943-8149/a000137

[35] König LM Sproesser G Schupp HT Renner B. Describing the process of adopting nutrition and fitness apps: behavior stage model approach. JMIR Mhealth Uhealth. (2018) 6(3):e55. 10.2196/mhealth.8261 29535078 PMC5871740

[36] R Core Team. R: A Language and Environment for Statistical Computing. Vienna, Austria: R Foundation for Statistical Computing (2025). Available online at: https://www.R-project.org (Accessed March 27, 2026).

[37] Schlottau S Cawello W Läer S. A novel model describing blood pressure profiles. Front Cardiovasc Med. (2025) 12:1–9. 10.3389/fcvm.2025.1583046 40547501 PMC12179137

[38] Kinny F Schlottau S Ali Sherazi B Obarcanin E Läer S. Digital health in pharmacy education: elective practical course integrating wearable devices and their generated health data. Exploratory Research in Clinical and Social Pharmacy. (2024) 15:100465. 10.1016/j.rcsop.2024.100465 38983639 PMC11231589

[39] Franklin SS O’Brien E Staessen JA. Masked hypertension: understanding its complexity. Eur Heart J. (2016) 38(15):1112–8. 10.1093/eurheartj/ehw502 27836914

[40] Bilo G Grillo A Guida V Parati G. Morning blood pressure surge: pathophysiology, clinical relevance and therapeutic aspects. Integr Blood Press Control. (2018) 11:47–56. 10.2147/IBPC.S130277 29872338 PMC5973439

[41] Lecarpentier Y Schussler O Hébert JL Vallée A. Molecular mechanisms underlying the circadian rhythm of blood pressure in normotensive subjects. Curr Hypertens Rep. (2020) 22(7):50. 10.1007/s11906-020-01063-z 32661611 PMC7359176

[42] Hahad O Rajagopalan S Lelieveld J Sørensen M Frenis K Daiber A. Noise and air pollution as risk factors for hypertension: part I-epidemiology. Hypertension. (2023) 80(7):1375–83. 10.1161/HYPERTENSIONAHA.122.18732 37073726 PMC10330192

[43] Zhang S Qian ZM Chen L Zhao X Cai M Wang C. Exposure to air pollution during Pre-hypertension and subsequent hypertension, cardiovascular disease, and death: a trajectory analysis of the UK biobank cohort. Environ Health Perspect. (2023) 131(1):17008. 10.1289/EHP10967 36696106 PMC9875843

[44] Leng B Jin Y Li G Chen L Jin N. Socioeconomic status and hypertension: a meta-analysis. J Hypertens. (2015) 33(2):221–9. 10.1097/HJH.0000000000000428 25479029

[45] Oude Griep LM Frost G Holmes E Wareham NJ Elliott P. Systems approach to investigate the role of fruit and vegetable types on vascular function in Pre-hypertensive participants: protocol and baseline characteristics of a randomised crossover dietary intervention. Nutrients. (2024) 16(17):2923. 10.3390/nu16172923 39275238 PMC11397325

[46] Savica V Bellinghieri G Kopple JD. The effect of nutrition on blood pressure. Annu Rev Nutr. (2010) 30:365–401. 10.1146/annurev-nutr-010510-103954 20645853

[47] John AT Chowdhury M Islam MR Mir IA Hasan MZ Chong CY. Effectiveness of high-intensity interval training and continuous moderate-intensity training on blood pressure in physically inactive Pre-hypertensive young adults. J Cardiovasc Dev Dis. (2022) 9(8):246. 10.3390/jcdd9080246 36005410 PMC9410224

[48] Li W Liu H Wang X Liu J Xiao H Wang C. Interventions for reducing blood pressure in prehypertension: a meta-analysis. Front Public Health. (2023) 11:1139617. 10.3389/fpubh.2023.1139617 37033077 PMC10078829

[49] Lu Y Lu M Dai H Yang P Smith-Gagen J Miao R. Lifestyle and risk of hypertension: follow-up of a young Pre-hypertensive cohort. Int J Med Sci. (2015) 12(7):605–12. 10.7150/ijms.12446 26283878 PMC4532965

[50] Palagini L Bruno RM Gemignani A Baglioni C Ghiadoni L Riemann D. Sleep loss and hypertension: a systematic review. Curr Pharm Des. (2013) 19(13):2409–19. 10.2174/1381612811319130009 23173590

[51] Gupta-Malhotra M Hashmi SS Barratt MS Milewicz DM Shete S. Familial aggregation of first degree relatives of children with essential hypertension. Blood Press. (2018) 27(5):289–96. 10.1080/08037051.2018.1463818 29699426 PMC6120796

[52] Kolber MR Scrimshaw C. Family history of cardiovascular disease. Can Fam Physician. (2014) 60(11):1016. 25392442 PMC4229162

[53] Niiranen TJ McCabe EL Larson MG Henglin M Lakdawala NK Vasan RS. Risk for hypertension crosses generations in the community: a multi-generational cohort study. Eur Heart J. (2017) 38(29):2300–8. 10.1093/eurheartj/ehx134 28430902 PMC6075041

[54] Wright CE O'Donnell K Brydon L Wardle J Steptoe A. Family history of cardiovascular disease is associated with cardiovascular responses to stress in healthy young men and women. Int J Psychophysiol. (2007) 63(3):275–82. 10.1016/j.ijpsycho.2006.11.005 17234292

[55] Li Y Godwin H Cecelja M O'Gallagher K Shah A Douiri A. Prediction of cardiovascular disease events from the photoplethysmograph waveform. J Am Heart Assoc. (2025) 0(0):e040237. 10.1161/JAHA.124.040237 PMC12826887 41334714

[56] Van Vliet M Monnink SHJ Kuiper MJ Constandse JC Hoftijzer D Ronner E. Evaluation of a novel cuffless photoplethysmography-based wristband for measuring blood pressure according to the regulatory standards. Eur Heart J Digit Health. (2024) 5(3):335–43. 10.1093/ehjdh/ztae006 38774367 PMC11104472

[57] Zanelli S Ammi M Hallab M El Yacoubi MA. Diabetes detection and management through photoplethysmographic and electrocardiographic signals analysis: a systematic review. Sensors (Basel. (2022) 22(13):4890. 10.3390/s22134890 35808386 PMC9269150

