# Supplementary material for: Continuous assessment and research on enhanced blood pressure control – CareBP study
Source: Front Cardiovasc Med. 2026 Aug 4;13:1857454. doi: 10.3389/fcvm.2026.1857454 (PMC13481507; doi:10.3389/fcvm.2026.1857454)
Supplement: Supplementary file 1 [file Datasheet1.pdf]

Supplementary Material

1 Supplementary Data

Questionnaire 1. Questionnaire about general and health-related information at the beginning of the study.

Heinrich Heine Universität Düsseldorf

Klinische Pharmazie & Pharmakotherapie

Welcome to the CareBP-Study general and health-related questionnaire!

Study ID: ______________________________

General

1. **How old are you in years?** _______________

2. **What is your gender?**
☐ Male ☐ Female ☐ Diverse ☐ Not specified

3. **How tall are you (in meters)?** _______________

4. **How much do you weigh (in kilograms)?** _______________

5. **Do you have pharmaceutical/ medical education?**
☐ Yes What kind? __________________________
☐ No

6. **Do you have experience with a cuffless blood pressure measurement device?**
☐ Yes → How did you gain the experience?
☐ in a private setting
☐ as part of medical care
☐ through participation in a previous study
☐ No

7. **Did you have already medical ambulatory blood pressure measurement (24h)?**
☐ Yes
☐ No

Continued from Questionnaire 1. Questionnaire about general and health-related information at the beginning of the study.

Heinrich Heine Universität Düsseldorf

Klinische Pharmazie & Pharmakotherapie

Health-related

1. Do you smoke or have you smoked in the past?

☐ Yes, I am a smoker
☐ Yes, I have smoked in the past → When was the last time? _______________
☐ No, neither currently nor previously

2. How active are you?

Work
☐ only sitting work
☐ sitting work, temporarily walking or standing
☐ predominantly walking and standing
☐ physically demanding

Free time
☐ little or no demanding activities
☐ irregularly moderate to demanding activities
☐ regularly moderate to demanding activities
☐ very high activity, high-performance sport

2b How many minutes per week are you active in your free time?

☐ < 75 min ☐ 75 - 150 min ☐ ≥ 150 min

Over how many days?

☐ 1-3 days ☐ 3-5 days ☐ 5-7 days

3. How is your diet?

☐ vegan
☐ vegetarian
☐ balanced with plant- and animal-based food
☐ predominantly animal-based food

3b Please be more specific about your diet.

	Strongly disagree	Disagree	Neither agree nor disagree	Agree	Strongly agree
I eat plenty of fruit and fresh vegetables.	☐	☐	☐	☐	☐
I pay attention to low-salt diet.	☐	☐	☐	☐	☐
I pay attention to low-fat diet.	☐	☐	☐	☐	☐
I pay attention to low-sugar diet.	☐	☐	☐	☐	☐
I eat no fast-food.	☐	☐	☐	☐	☐
I eat no convenience food.	☐	☐	☐	☐	☐
I drink no alcohol.	☐	☐	☐	☐	☐

4. Has high blood pressure been diagnosed in one or more members of your family?

☐ Yes → ☐ Parents ☐ Grandparents ☐ Siblings
☐ No
☐ Unknown

5. Have any members of your family (one or more) ever suffered from cardiovascular events such as heart attacks or strokes?

☐ Yes → ☐ Parents ☐ Grandparents ☐ Siblings
☐ No
☐ Unknown

Questionnaire 2. Satisfaction questionnaire at the end of the study

Heinrich Heine Universität Düsseldorf

Klinische Pharmazie & Pharmakotherapie

Welcome to the CareBP-Study satisfaction questionnaire!

Study ID: ______________________________

1. How much do you agree with the following statements to the satisfaction with the blood pressure bracelet?

	Strongly disagree	Disagree	Neither agree nor disagree	Agree	Strongly agree
The positioning of the bracelet went flawlessly.	☐	☐	☐	☐	☐
The calibration with the cuff went flawlessly.	☐	☐	☐	☐	☐
The bracelet had an impact on my daily routines.	☐	☐	☐	☐	☐
I found wearing the bracelet comfortable during the day.	☐	☐	☐	☐	☐
While wearing the bracelet, I was able to perform everyday tasks without restriction.	☐	☐	☐	☐	☐
I found wearing the bracelet comfortable during the night.	☐	☐	☐	☐	☐
The app was easy to use.	☐	☐	☐	☐	☐
Synchronizing the values with the cloud went flawlessly.	☐	☐	☐	☐	☐
I was able to get the information I needed from the app.	☐	☐	☐	☐	☐
The time required to use the app was appropriate.	☐	☐	☐	☐	☐
I am satisfied with the bracelet as blood pressure measurement tool.	☐	☐	☐	☐	☐
I would use the bracelet again for another monitoring.	☐	☐	☐	☐	☐

2. How much do you agree with the following statements to awareness and perception of blood pressure?

	Strongly disagree	Disagree	Neither agree nor disagree	Agree	Strongly agree
I was able to develop an awareness for my blood pressure values.	☐	☐	☐	☐	☐
The study participation has been personally rewarding for me.	☐	☐	☐	☐	☐
The blood pressure monitoring helped me to better understand blood pressure changes.	☐	☐	☐	☐	☐
I would like health insurance to reimburse the costs of continuous blood pressure monitoring.	☐	☐	☐	☐	☐
I think the involvement of pharmacists in blood pressure monitoring is important.	☐	☐	☐	☐	☐
I have planned to continue blood pressure measurements (independently of the measurement method).	☐	☐	☐	☐	☐

3. Further comments?

2 Supplementary Figures and Tables

2.1 Supplementary Figures

Supplementary Figure 1. Blood pressure profiles of six normotensive adults (A-F) from already published data

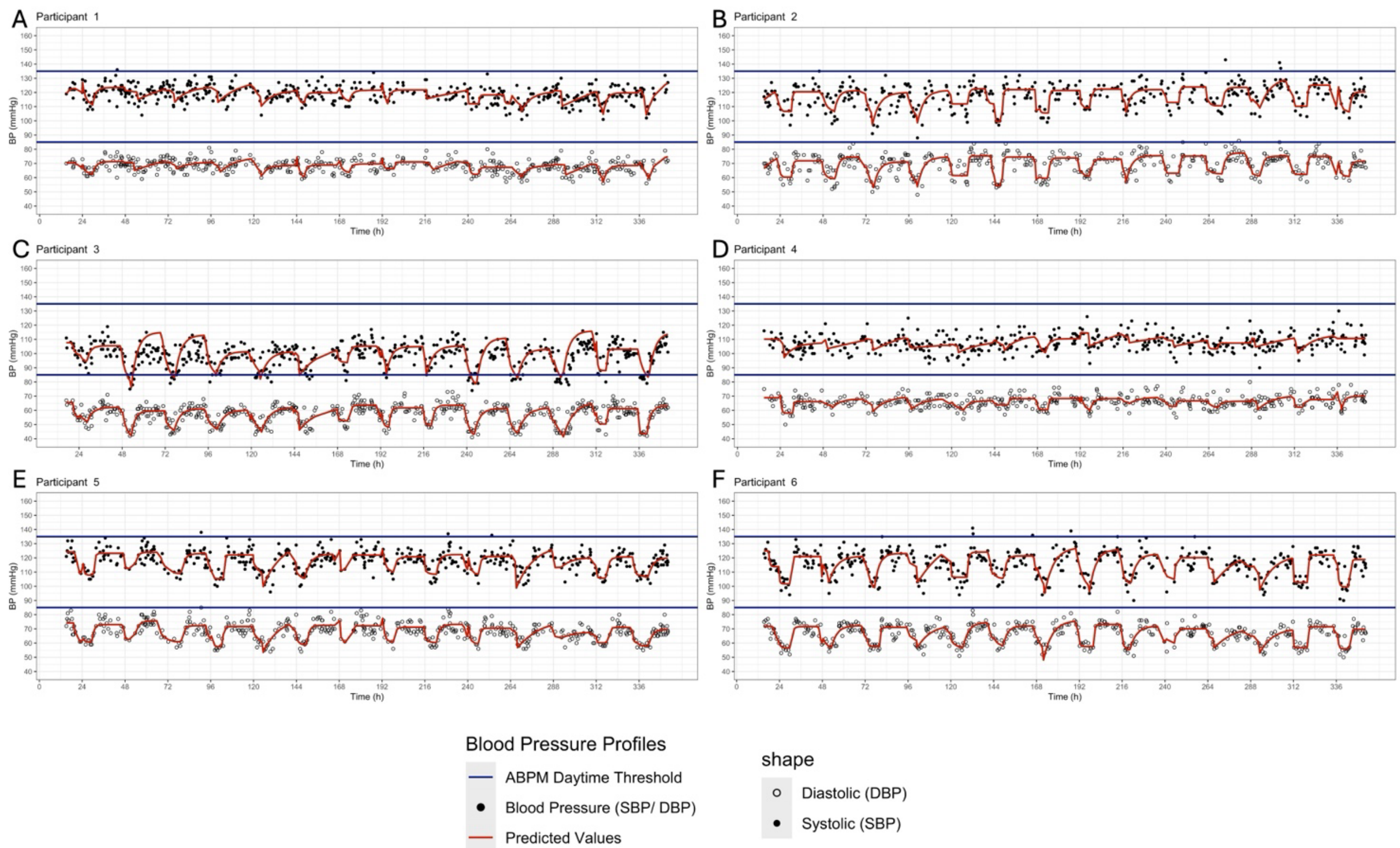

Reference: Schlottau S, Cawello W, Läer S. (2025). A novel model describing blood pressure profiles. Frontiers in Cardiovascular Medicine, Volume 12 - 2025. https://doi.org/10.3389/fcvm.2025.1583046

Supplementary Figure 2. Systolic Blood Pressure Model - Plot of the weighted residuals (%) for all ten participants (A-J) in the shape of blue squares against time (h).

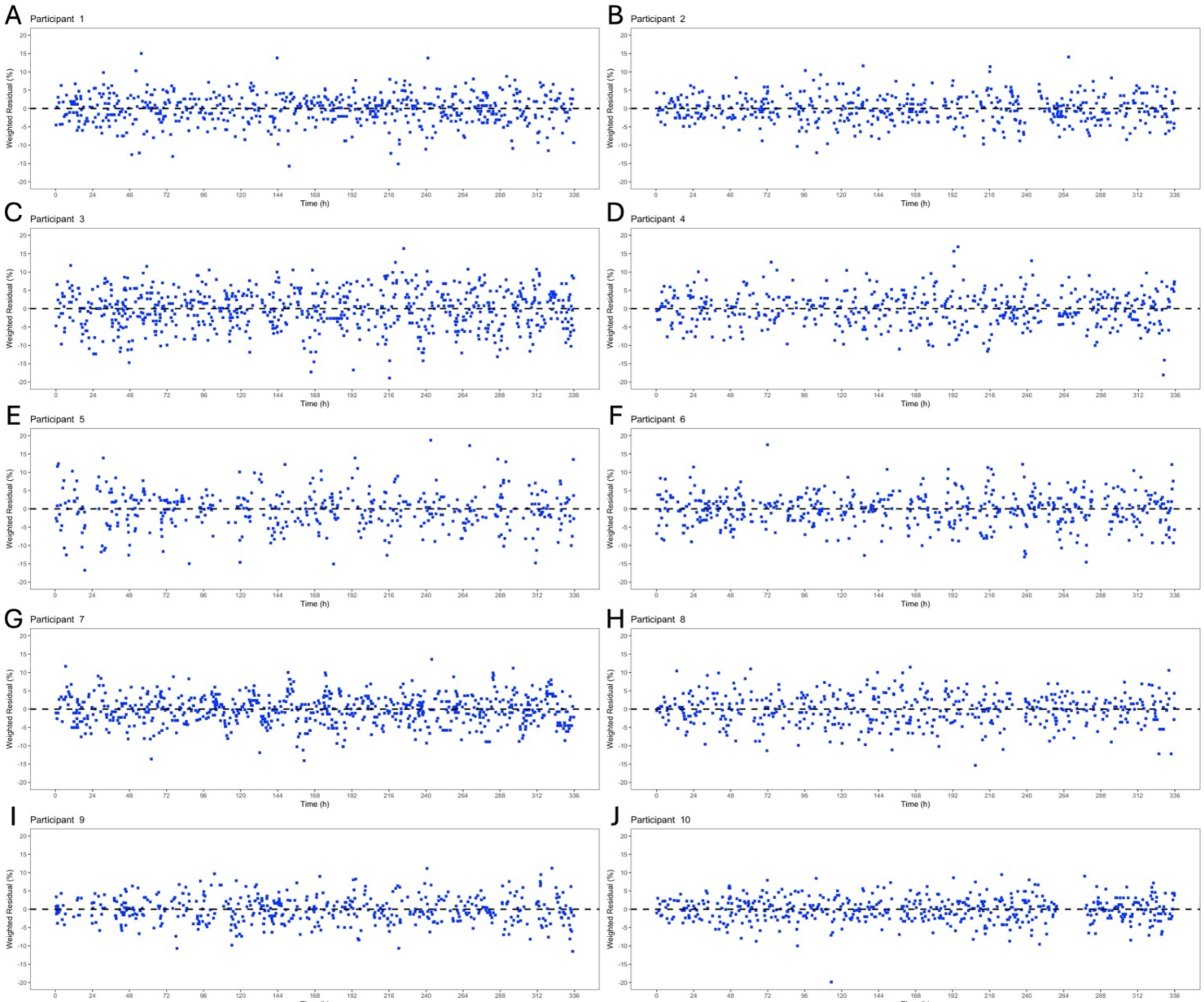

Supplementary Figure 3. Diastolic Blood Pressure Model - Plot of the weighted residuals (%) for all ten participants (A-J) in the shape of blue squares against time (h).

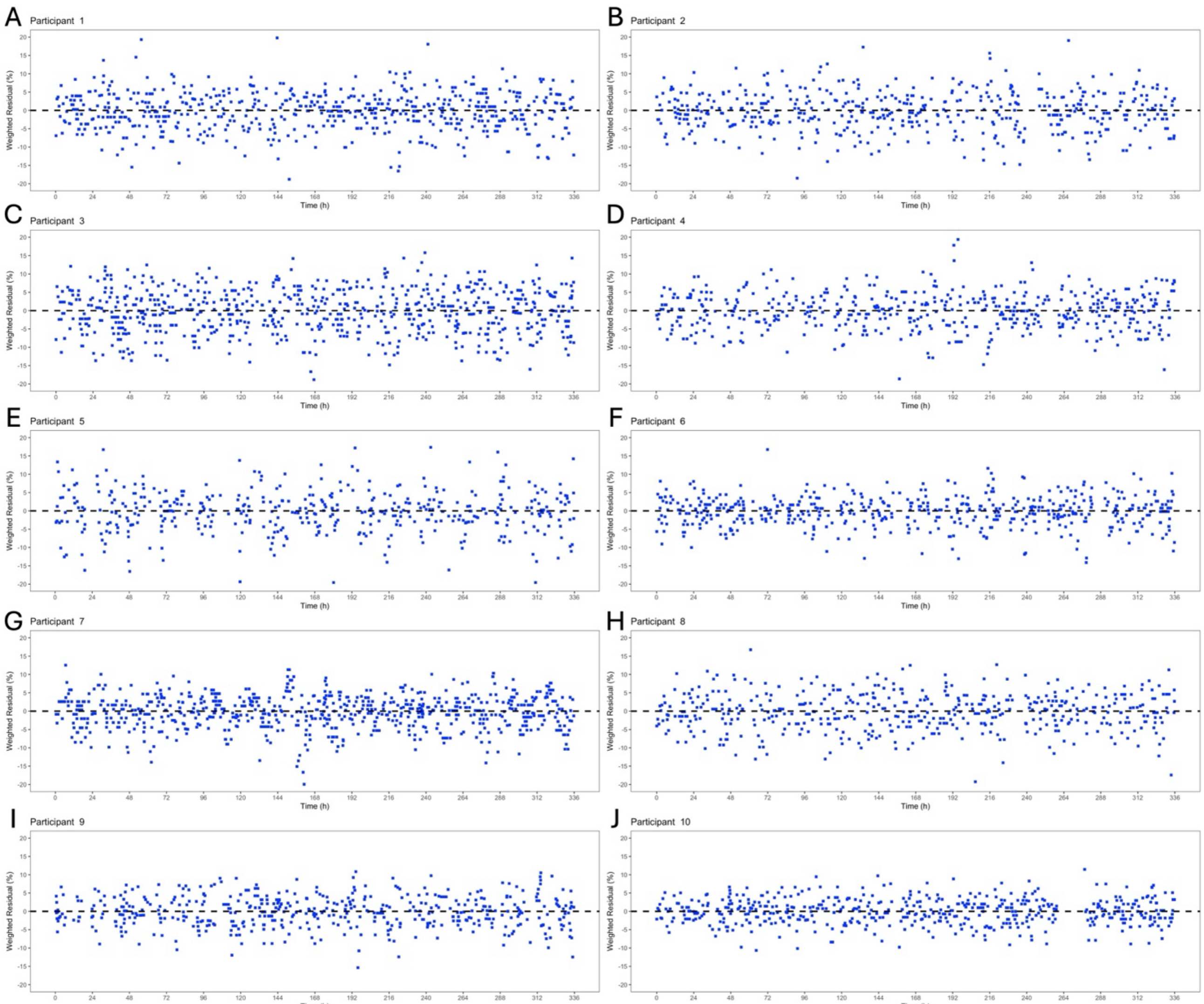

2.2 Supplementary Tables

Supplementary Table 1. Diet of the participants (questionnaire 1)

#	Statement	Mean (±SD)	95% CI
1	I eat plenty of fruit and fresh vegetables.	3.30 (±0.82)	2.50 - 4.10
2	I pay attention to low-salt diet.	2.80 (±1.40)	1.43 - 4.17
3	I pay attention to low-fat diet.	2.80 (±0.79)	2.03 - 3.57
4	I pay attention to low-sugar diet.	3.00 (±1.25)	1.78 - 4.23
5	I eat no fast-food.	3.00 (±1.05)	1.97 - 4.03
6	I eat no convenience food.	2.30 (±0.82)	1.50 - 3.10
7	I drink no alcohol.	3.60 (±0.97)	2.65 - 4.55

Supplementary Table 2. Perception of participants regarding blood pressure (questionnaire 2)

#	Statement	Mean (±SD)	95% CI
1	I was able to develop an awareness for my blood pressure values.	4.50 (±0.53)	3.98 - 5.02
2	The study participation has been personally rewarding for me.	4.70 (±0.48)	4.23 - 5.17
3	The blood pressure monitoring helped me to better understand blood pressure changes.	3.70 (±0.67)	3.04 - 4.36
4	I would like health insurance to reimburse the costs of continuous blood pressure monitoring.	4.40 (±0.70)	3.71 - 5.09
5	I think the involvement of pharmacists in blood pressure monitoring is important.	4.80 (±0.42)	4.39 - 5.21
6	I have planned to continue blood pressure measurements (independently of the measurement method).	4.70 (±0.48)	4.23 - 5.17
